# The Microscopic Mechanism and Rheological Properties of SBS-Modified Asphalt with Warm Mixing Fast-Melting

**DOI:** 10.3390/ma16165690

**Published:** 2023-08-19

**Authors:** Weiguang Huo, Yazhou Zhuang, Ziran Wang, Xiaolong Kang, Riran Wang

**Affiliations:** 1 Zhengzhou Public Utility Investment Development Group Co., Ltd., Zhengzhou 450016, China; 2 Yellow River Laboratory, Zhengzhou University, Zhengzhou 450001, Chinazzukangxiaolong@163.com (X.K.)

**Keywords:** fast-melting, viscoelastic characteristic, performance of fatigue, modification mechanism

## Abstract

To overcome the shortcomings of traditional wet styrene-butadiene-styrene (SBS) modification technology, such as its high energy consumption and thermal decomposition, a warm mix and fast-melting SBS modifier was developed. Based on the theory of rheology, a dynamic shear rheometer (DSR) was applied to investigate the viscoelastic properties of the warm mix and fast-melting SBS-modified asphalt using a frequency scanning test. Atomic force microscopy (AFM) was used to reveal the modification mechanism of the SBS-modified asphalt. An investigation of the thermal stability of the asphalt binder was conducted using a thermogravimetric test (TG). The results exhibited that the SBS-modified asphalt had good viscoelastic properties, as well as thermal stability. The “bee structure” of the SBS-modified asphalt was finer and more stable. In addition, the adhesion and the Derjaguin–Muller–Toporov (DMT) modulus of the SBS-modified asphalt at a warm mixing speed was higher than that of regular SBS-modified asphalt.

## 1. Introduction

With economic development, countries around the world have further increased their investments in transportation infrastructure, and the construction of a large number of high-grade highways has gradually become an urgent need [1]. High-grade highways carry a higher level of traffic than those before. As a result, the requirements for road materials are more stringent, and general asphalt mixtures already struggle to meet the requirements [2]. The emergence of asphalt modification technology is a good solution to this problem. With the help of various modifiers, modified asphalt has excellent properties [3]. Among the currently available modifiers, styrene-butadiene-styrene triblock copolymers (SBS) are the most prominently used in the field of modified asphalt. SBS-modified asphalt has a wide range of service temperatures, such as a good resistance to permanent deformation at high temperatures and a good resistance to fatigue cracking at low temperatures. The use of SBS-modified asphalt has been recognized as a good alternative to conventional modifiers [4]. The amount of SBS modifier used is a key factor affecting the performance of SBS-modified asphalt. The higher the amount of SBS is, the more conducive this is to the high performance of the asphalt binder [5].

The majority of roads built throughout the world use SBS-modified asphalt pavement, which is a conventional pavement structure with an excellent performance. However, the conventional wet SBS modification method has technical flaws such segregation and thermal disintegration, in addition to significant energy usage [6]. To alleviate this situation, the traditional wet modification process uses a high-speed shear to break up the SBS and disperse it into the asphalt. The process, though, suffers from technical shortcomings. Technical defects are revealed as follows. (1) After a high-speed shear, SBS-modified asphalt will display an SBS precipitation phenomenon. This phenomenon affects the stability of modified asphalt and will be exacerbated with an extension of storage time. (2) The persistent high-temperature environment throughout the modified asphalt transportation process ages the asphalt and damages the structural integrity of the SBS. (3) Preparing modified asphalt via a wet modification procedure takes 2–3 h of high-temperature shearing, swelling, and development [7]. Consequently, conventional wet modification techniques require the consumption of large amounts of fuel to maintain a high-temperature environment. This leads to huge energy consumption and produces greenhouse gases, leading to negative impacts on the environment [8,9,10]. Dry SBS-modified technology (SBS-T) reduces the repeated transport of asphalt and improves the durability of asphalt surfaces without increasing the cost by combining SBS-modified asphalt processing with the asphalt concrete mixing production process, thus bringing about significant economic and social benefits [11]. Therefore, dry SBS-modified asphalt technology has broad prospects for development. The promotion of dry SBS asphalt modification technology is also in response to the call of the state and provides good technical support for the construction of industrial green highways.

Conventional hot-mix asphalt (HMA) is produced at temperatures higher than 140 °C. Such a high-temperature production environment increases fuel costs, harmful smoke emissions, and hazardous gas emissions [12]. In recent decades, industries have been working to reduce environmental pollution, and researchers of asphalt materials are no exception [13,14]. Therefore, researchers have been exploring technology that meets the requirements of material properties and construction performance while reducing production energy and fuel consumption. Warm mix technology (WMT) has been proven to fulfill both of these requirements. According to the world ban, fuel exhaustion decreases by approximately 1 L and corresponding CO_2_ emissions decrease by 1 kg/ton for every 10 °C decrease in the temperature at which asphalt is produced. WMA has been frequently utilized to build asphalt pavements with a wide variety of production temperatures among the aforementioned technologies [15,16]. When compared to HMA (150–170 °C), WMA may be generated and built at lower temperatures (100–140 °C) without degrading the mix’s performance [17]. The use of WMT is effective in reducing harmful emissions and hazardous fumes [18,19]. In addition to the benefits of lower temperatures, the use of WMA improves workability and increases transportation distances. In addition, reducing the production temperature decreases the energy consumption, which, in turn, reduces production costs [20,21].

Warm mix dry SBS modification technology can further reduce the energy consumption of SBS-modified asphalt and its mixture in the production and construction process, as well as harmful gas emissions. This is due to dry SBS asphalt modification technology having some advantages in terms of energy savings and emission reduction [22]. Guolu Gaoke Engineering Technology Institute Co., Ltd. (Beijing, China) has effectively created a warm-melting SBS modifier (SBS-W) based on the research and development system of a fast-melting SBS modifier by fusing small-molecule SBS with organic viscosity reduction technology [23,24,25]. The industry used warm mixing and SBS modification integration technology for the first time.

In this paper, based on the existing rapid-melting SBS asphalt modification technology, different types of asphalt materials with Sasobit were combined. The viscoelastic properties and microscopic modification mechanism of the hot mix rapid-melting SBS-modified asphalt were systematically investigated. This study can serve as a technological foundation for further enhancing dry SBS modification technology’s impact on asphalt pavement’s structural durability, energy savings, and emission reduction, thus facilitating dual-carbon development strategies in road construction [26].

## 2. Materials and Test Methods

### 2.1. Materials

The physical characteristics of the base asphalt binder are given in Table 1. Sasobit was adopted as a warm mix agent, and polymers—SBS, SBS-T, and SBS-W—were accepted as polymer modifiers. Henan Lupeng Transportation Technology (Zhengzhou, China) provided the Sasobit modifier. The technical details are displayed in Table 2. Guolu Gaoke Engineering Technology Institute Co., Ltd. (Beijing, China) supplied the SBS, SBS-T, and SBS-W, as well as their parameters. Table 3 provides details on each modification.

### 2.2. Preparation of Sample

Three different types of polymer-modified asphalt (PMA) samples were prepared using additives of 5% SBS, 5% SBS-T, and 6% SBS-W. A total of 3% Sasobit was added as a warm mixing agent to the three types of polymer-modified asphalt. The base asphalt was completely melted in an oven at 120 °C. Then, the SBS, SBS-T, and SBS-W modifiers were added to the base asphalt. After that, the mixtures were sheard at a speed of 5000 rpm for 30 min at 170 °C using a high-speed shear machine. After the PMA’s preliminary stage was finished, 3% Sasobit warm mix was added and blended for 30 min on a high-speed shearing machine at 5000 r/min. The remaining PMA was then heated in an oven for 30 min at 140 °C.

### 2.3. Aging Methods

In this study, the asphalt initially underwent a rotating film oven test (RTFOT, ASTM D2872 [27]) for short-term laboratory aging. After the RTFOT test, PAV and UV aging were applied to the asphalt residue. The RTFOT residue was applied to a stainless steel plate in accordance with the test’s requirements for the thickness of the asphalt film. Then, it was placed within a pressure vessel with 2.1 MPa of air pressure and aged for 20 h at the chosen aging temperature of 100 °C. In the end, the residue was vacuum degassed.

The UV aging test has a unique operating technique that involved taking a 20 g modified asphalt sample after brief aging and pouring it into a stainless steel dish with a diameter of 140 mm and depth of 10 mm. The asphalt film could be created with a thickness of about 1 mm, allowing UV light to be uniformly irradiated on the asphalt film, ensuring the impact of ultraviolet aging. It was automatically leveled and coated with an aging dish in the oven. The modified asphalt sample was then put into the aging tray and set on the sample rack within the UV aging chamber. The rotation speed was set to 5 r/min, the test temperature to 45 ± 0.5 °C, and the UV radiation instrument’s radiation intensity measurement to 140 w/m^2^. Seven days passed during the UV exposure. The aging processes were completed in accordance with AASHTO TP101.

### 2.4. Microscopic Characteristics

#### 2.4.1. Atomic Force Microscopy (AFM)

Nanoscale surface features can be described using AFM. AFM is being increasingly employed for microanalyses of the composition and generation process of honeycomb structures in modified asphalt, since an increase in asphaltene concentration may be associated with an increase in the density of the honeycomb microstructures on the surface of asphalt. The corrugated microstructures that appear in the AFM images of asphalt are what we refer to as “honeycomb structures” and are depicted as yellow and black stripes. The “honeycomb structure” can be used to symbolize the four parts of asphalt, and the forces that hold these four parts together can be characterized as adhesion. As a result, modifications to the “honeycomb structure” will alter the mechanical characteristics of asphalt, which will alter the performance of modified asphalt on large roads. In addition, this “honeycomb structure” can rationally explain the development of the law, as well as the modification mechanisms of modifiers on asphalt, providing a theoretical framework for the study.

In order to investigate the impacts of various modifiers on matrix asphalt, atomic force microscopy was utilized in this work to examine the surface morphology and micromechanical characteristics of various modified asphalt cement samples in light percussion mode. The microscope probe used was the Bruker Tap 150A probe (Billerica, MA, USA). The resonance frequency was 75 kHz, the radius was 20 nm, the scanning frequency was 1 Hz, the scanning range was 20 × 20 μm, the number of scanning points was 256 × 256 in the scanning range, and the stiffness was 5 N/m.

#### 2.4.2. Thermogravimetric (TG) Test

By evaluating the relationship between the asphalt mass change and temperature, a thermogravimetric analysis can be used to determine the effect of material qualities and composition on the thermal stability of modified asphalt. The TG test can also be used to look into the temperature range and mass loss rate of asphalt materials during thermal decomposition. The thermogravimetric test findings are displayed as thermogravimetric curves (TG curves), which show how mass m and temperature T relate to one another.

To test the thermal stability of modified bitumen, a thermogravimetric test was performed in this study using a synchronous thermal analyzer (STA449F3 Jupiter) produced by NETZSCH, Selb, Germany, under the following conditions: a sample mass of 10 mg, a nitrogen atmosphere, a test temperature scanning range of 30–750 °C, and a heating rate of 20 °C/min. Following the weighing of 10 mg of modified bitumen samples using a highly accurate balance, the samples were then put into the test device’s crucible before the system was filled with nitrogen and the heating rate was adjusted. The test was conducted as follows: a modified asphalt sample weighing approximately 10 mg was weighed using a high-precision balance before being placed in the instrument’s crucible, followed by a thorough vacuuming of the system before nitrogen was added and the rate of temperature rise was adjusted. The TG tests were carried out according to ASTM E2550 [28].

### 2.5. Frequency Scan (FS) Test

Without causing any damage to the test specimens, the FS test can be performed to ascertain the mechanical parameters of the viscoelastic characteristics of asphalt pavements. It can also describe the viscoelastic characteristics of improved asphalt pavements under various loading regimes and temperatures. The linear dynamic viscoelastic modulus |G*|_LVE_ of modified asphalt under fatigue test loading conditions can be calculated by building the dynamic shear modulus (G*) master curve in accordance with the time–temperature equivalence principle.

A dynamic shear rheometer was employed in this investigation to choose a frequency range from 0.1 rad/s to 100 rad/s at 15 °C, 25 °C, and 35 °C. The strain control mode and 0.1% strain level were used to perform frequency sweep tests (FS) on the warm mix polymer-modified asphalt with six mixtures of original, pressurized aging vessel (PAV)−aged, and ultraviolet (UV)−aged. The main curve of the phase angle of the modified asphalt was fitted according to the data. Three parallel tests were conducted for each test to ensure the accuracy of the data, and the average value of the test data was calculated for subsequent analysis.

## 3. Results and Discussion

### 3.1. Topography of Asphalt Binders

**Figure 1:**
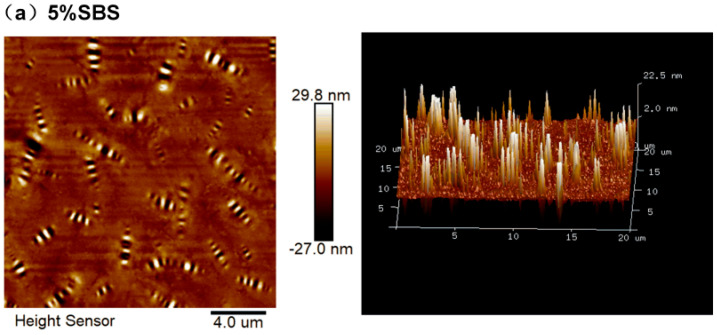

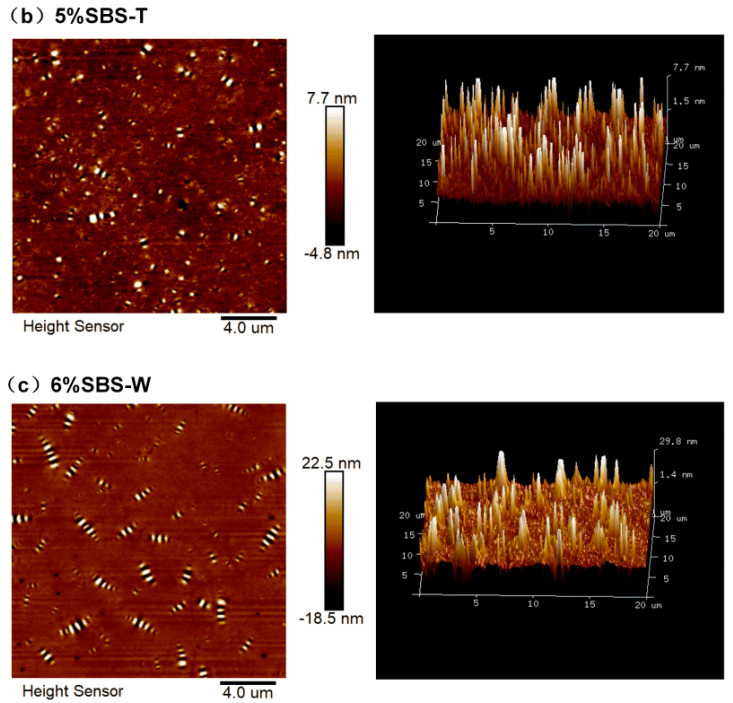
Two-dimensional atomic diagram and 3D topography of modified asphalt: (**a**) SBS; (**b**) SBS-T; and (**c**) SBS-W.

Figure 1 indicates that the amount of black and white striped “bee structures” in the fast-melting SBS (SBS-T, SBS-W)−modified asphalt binder gradually rose, while the area of the “bee structure” decreased when compared to the SBS-modified asphalt. In other words, the “bee structure” of the SBS-modified asphalt binder that melted quickly was finer. This was due to the rapid-melting modifier’s contribution to the formation of a more stable network structure of the asphalt system, which also served to improve the modified asphalt’s fatigue resistance by reducing the free movement and diffusion of the asphalt molecules. As can be seen from the 3D topography, there are more bright white areas and lower bright white wave peaks in the “mountains” of the SBS-modified asphalt, which also indicates that the system of the SBS-modified asphalt was more stable and had a better anti-fatigue performance.

**Figure 2:**
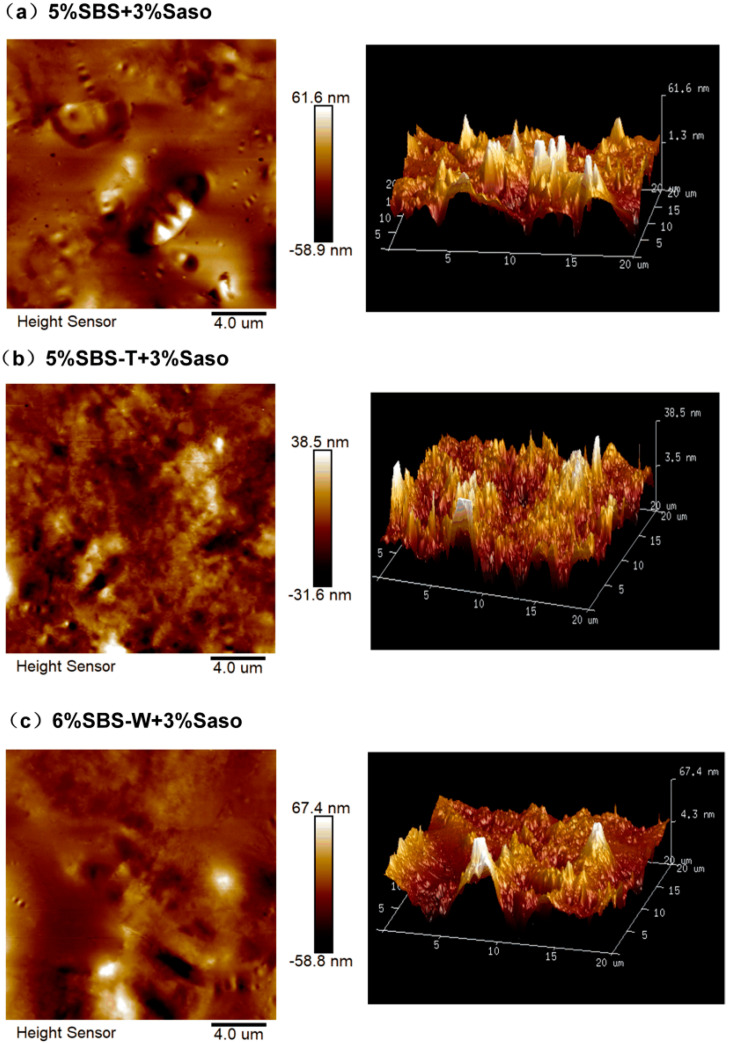
Two-dimensional atomic diagram and 3D topography of modified asphalt. (**a**) SBS/Saso; (**b**) SBS-T/Saso; and (**c**) SBS-W/Saso.

Figure 2 manifests a two-dimensional atomic diagram and the 3D topography of the warm-mixed polymer-modified asphalt obtained using the Nanoscope Analysis software v180r1. It is found from the image that the warm-mixed modified asphalt did not have a complete bee structure after the addition of Sasobit, and the fluctuation degree of the asphalt surface increased. Moreover, there are large color differences between different areas, and the “bee structure” of the SBS-modified asphalt with warm mixing speed melting is more dispersed and broken. The results demonstrate that the fatigue resistance of the SBS-modified asphalt with warm mixing speed melting was better [29,30].

The roughness index can be used to measure the microstructural data of various asphalts in order to objectively examine the variation in asphalt microphase states. Table 4 provides the specific data.

According to the table, the addition of Sasobit increased the root mean square roughness and indicated average roughness of the asphalt binder, and also increased the difference between the “honeycomb structure” phase of the asphalt and the “non-honeycomb structure” phase. This suggests that the average surface roughness of SBS-WMA was approximately 2.15 times higher than that of SBSMA, and that the SBS-W modifier increased the difference between the “honeycomb” and “non-honeycomb”. The results indicate that, when compared to the regular SBS modifier, the SBS-W modifier significantly widened the gap between the “honeycomb structure” phase and the “non-honeycomb structure” phase of the asphalt, i.e., the effect of increasing the number of “honeycomb structures” was more apparent, making the asphalt system of SBS-WMA more stable and achieving a better fatigue resistance. The modified asphalt that contained 6% SBS-W + 3% Saso had the highest roughness, which suggests that it had the best fatigue resistance.

### 3.2. Analysis of Asphalt Nano Adhesion

**Figure 3:**
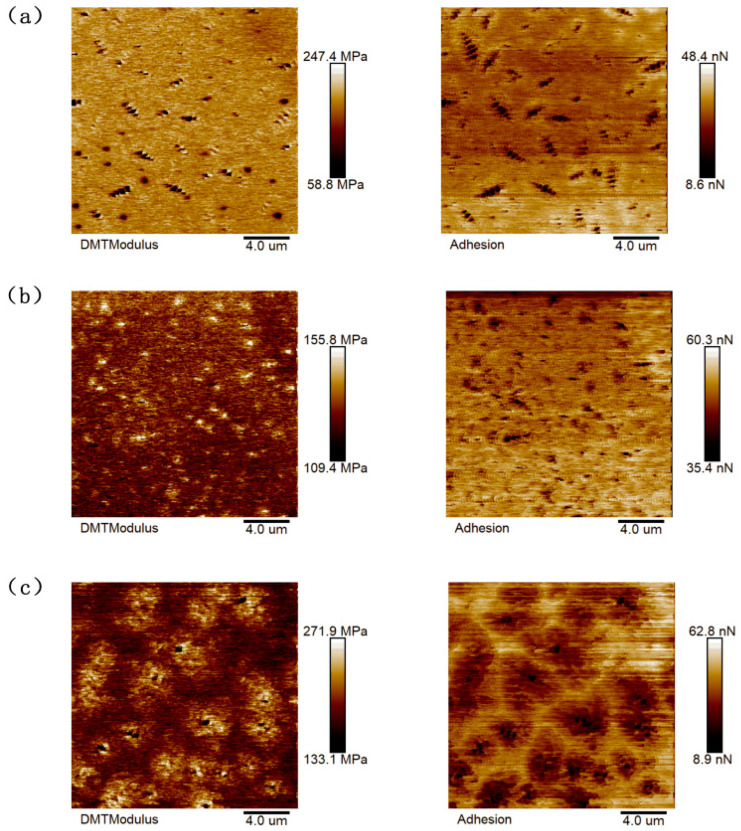
AFM mechanical image of modified asphalt: (**a**) SBS; (**b**) SBS-T; and(**c**) SBS-W.

**Figure 4:**
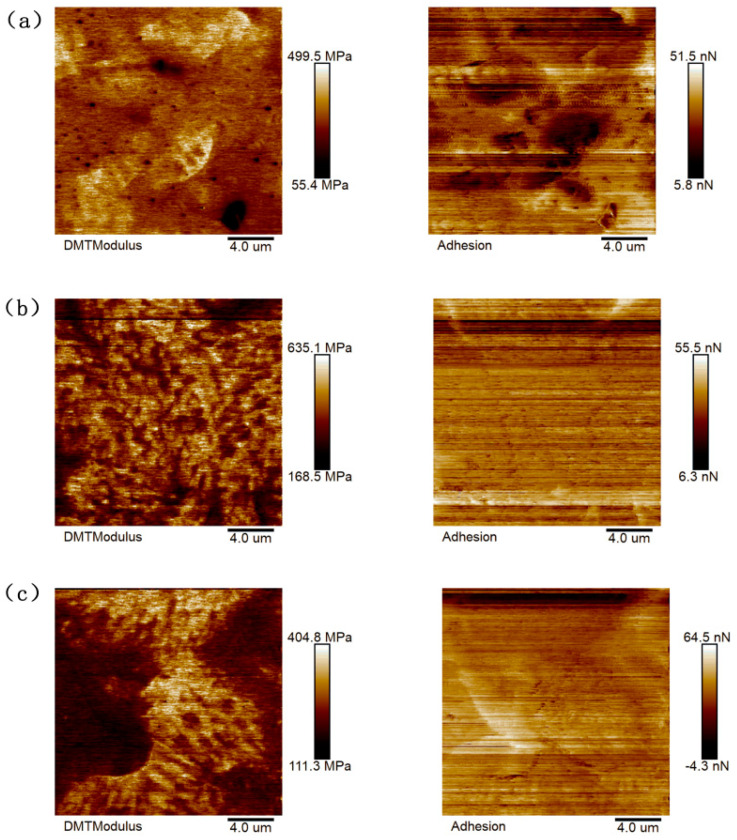
AFM mechanical image of modified asphalt: (**a**) SBS/Saso; (**b**) SBS-T/Saso; and (**c**) SBS-W/Saso.

Based on the results of scanning the asphalt samples with an atomic force microscope, the NanoScope Analysis software used with AFM could be imported to calculate the nano adhesion between the probe and the sample, and the statistical histogram of the adhesive force-distribution frequency could be derived, as shown in Figure 3 and Figure 4.

**Figure 5:**
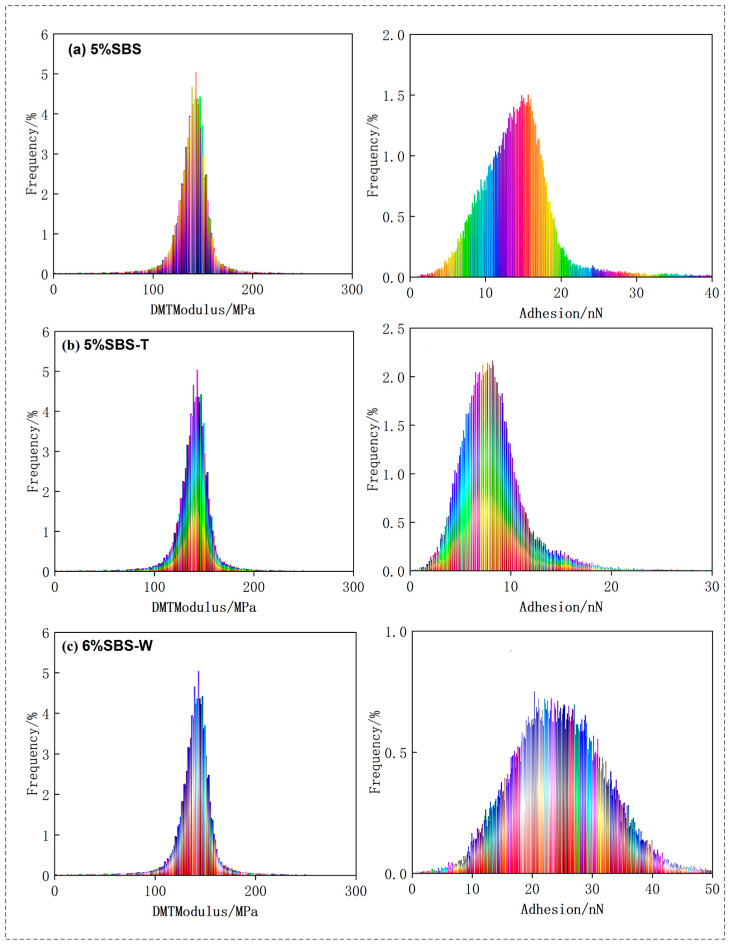
Histogram of DMT modulus and adhesion data distribution of modified asphalt. (**a**) SBS; (**b**) SBS-T; and (**c**) SBS-W.

**Figure 6:**
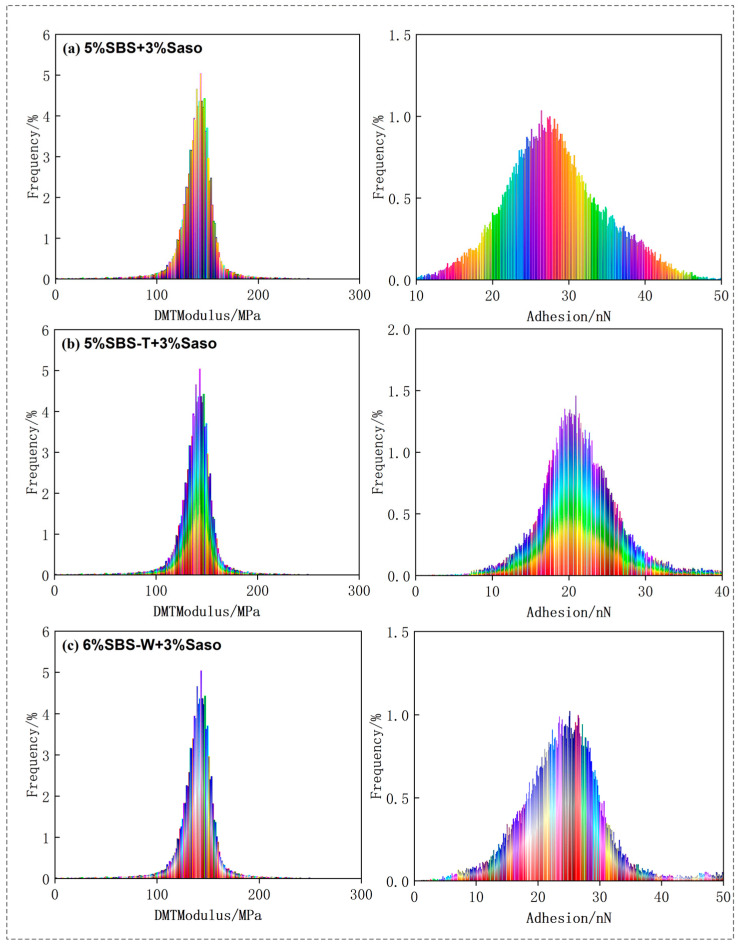
Histogram of DMT modulus and adhesion data distribution of warm mix modified asphalt. (**a**) SBS + Saso; (**b**) SBS-T + Saso; and (**c**) SBS-W + Saso.

The “honeycomb structure” of the quick-melt SBS-modified asphalt was more precisely tailored, as can be seen in the figure, leading to greater DMT modulus and adhesion values. With the Sasobit added, the warm-mixed quick-melt modified asphalt had a higher DMT modulus and adhesion value than the conventional warm-mixed SBS-modified asphalt. The data collected in the figure were analyzed using the Origin software 2021, and Figure 5 and Figure 6 were created to show the results. This allowed for a more quantitative evaluation of the DMT modulus and adhesion of the six asphalt cements.

It can be seen from the figure that the DMT modulus value and adhesive force value of the asphalt binder first increased and then decreased, approximating a normal distribution. The DMT modulus and adhesion values were calculated and quantitatively analyzed. Asphalt that has undergone SBS modification typically has a DMT modulus of 123.52 Mpa and an adhesive force of 22.25 nN. The SBS-W-modified asphalt had an average DMT modulus of 141.12 Mpa and an average adhesion of 29.26 nN. These two numbers, which are roughly 1.33 times and 1.16 times higher than those of regular SBS-modified asphalt, show that the addition of an SBS-W modifier improved the anti-deformation ability and stiffness of asphalt binder, which was reflected in a macro better anti-fatigue ability. As can be seen from Figure 5, the average DMT modulus value of the SBS-modified asphalt in the warm mix was 257.33 Mpa and the average adhesion value was 25.58 nN. The average DMT modulus value and average adhesion value of the SBS-T-modified asphalt were 328.40 Mpa and 28.31 nN, respectively. The average DMT modulus value and average adhesion value of the SBS-W-modified warm mix asphalt were 289.39 Mpa and 29.91 nN, respectively [31]. These two values of the SBS-W-modified warm mix asphalt are about 1.28 times and 1.11 times that of ordinary SBS-modified asphalt, indicating that the SBS and Sasobit had a better composite modifying effect, with a higher strength and better fatigue resistance.

### 3.3. Thermal Stability of Asphalt Binders

**Figure 7:**
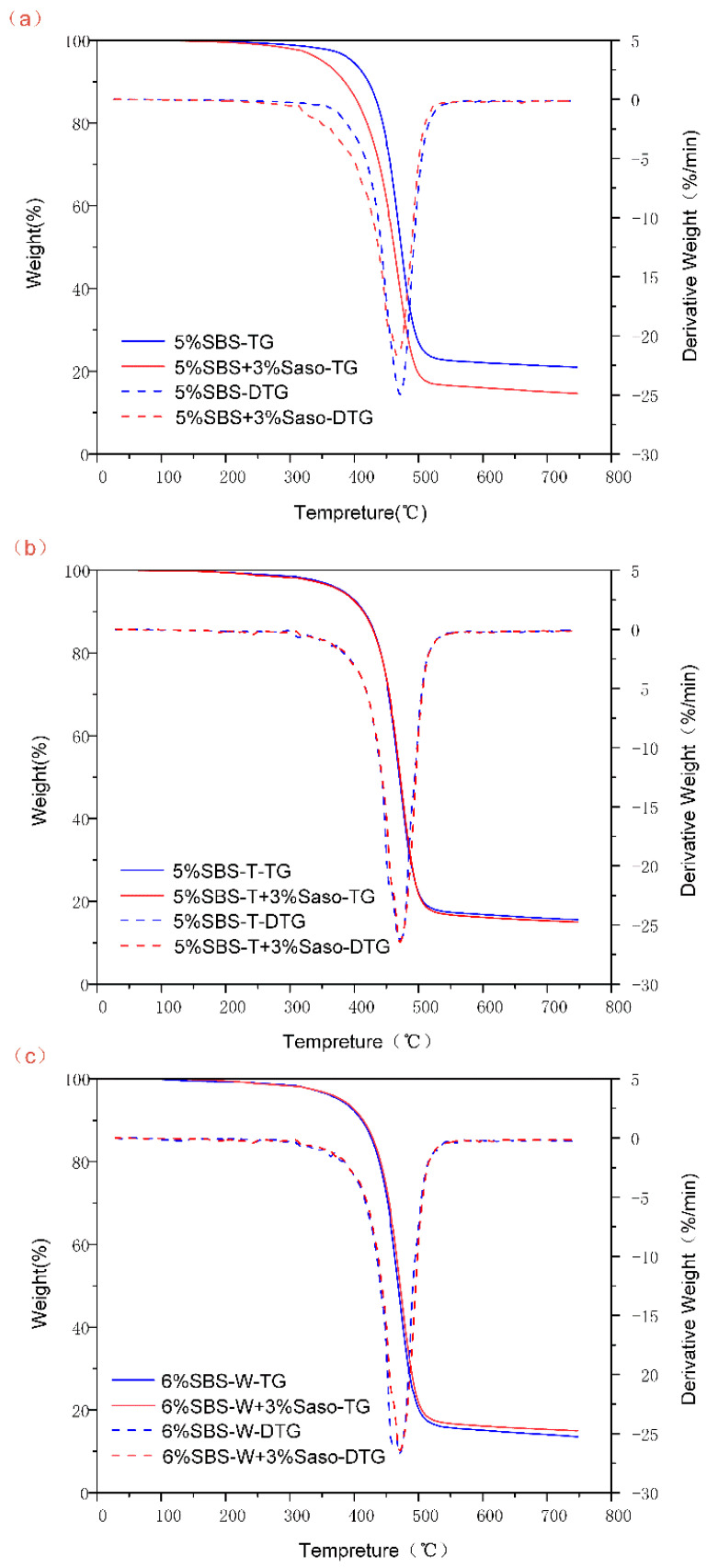
TG and DTG curves of modified asphalt binders: (**a**) Original asphalt, (**b**) PAV aging, and (**c**) UV aging.

As observed in Figure 7, the six different types of modified asphalt curve graph trends are fairly similar, and the quality change was mainly divided into three stages: this was mainly related to the asphalt material’s light component volatilization and asphaltene and colloidal combustion; when the temperature was higher than 500 °C, the quality of the modified asphalt changed in a smaller way, and at this point, the TG and derivative thermogravimetry (DTG) curves started to gradually tend to flake. The quality of the modified asphalt decreased significantly, with a quality loss rate of more than 80%, in the temperature range from 300 °C to 500 °C. This was primarily due to asphalt material in the volatilization of lightweight components and the combustion of gums and asphaltene, while at the same time, the asphaltene burned. This showed that the modified asphalt could maintain a high thermal stability in this temperature range. This was primarily caused by the asphalt material’s light constituents volatilizing, the gum and asphaltene burning, and the disintegration and cleavage of the bitumen binder’s big and small molecule components. In relation to the carbonization of asphalt under high-temperature settings, the quality of the modified asphalt varied less when the temperature exceeded 500 °C, and the TG and DTG curves gradually flattened.

Table 5 displays the T_max_ (the temperature at maximum breakdown rate), IDT (the temperature at 5% mass loss, also known as the initial decomposition temperature), and asphalt mass residual rate at 600 °C for the six polymer-modified asphalts. The IDTs of the three modified asphalts were all near to each other and hovered around 380 °C, as shown in Table 5. SBSMA had a comparatively high IDT.

When comparing the T_max_ of the various modified asphalt samples, it can be seen that SBS-WMA had the highest maximum decomposition rate at the corresponding temperature, indicating that the warm mixing fast-melting SBS-modified asphalt had a higher thermal stability when the temperature was lower than 500 °C. This was because the SBS-W modifier prevented the volatilization of the lightweight components of the decomposition of both small molecules and macromolecules within the asphalt.

### 3.4. Phase Angle Principal Curve

**Figure 8:**
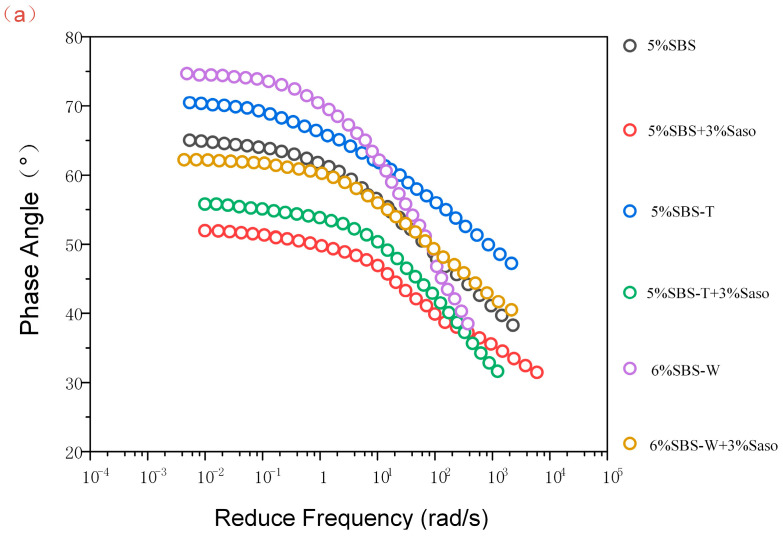

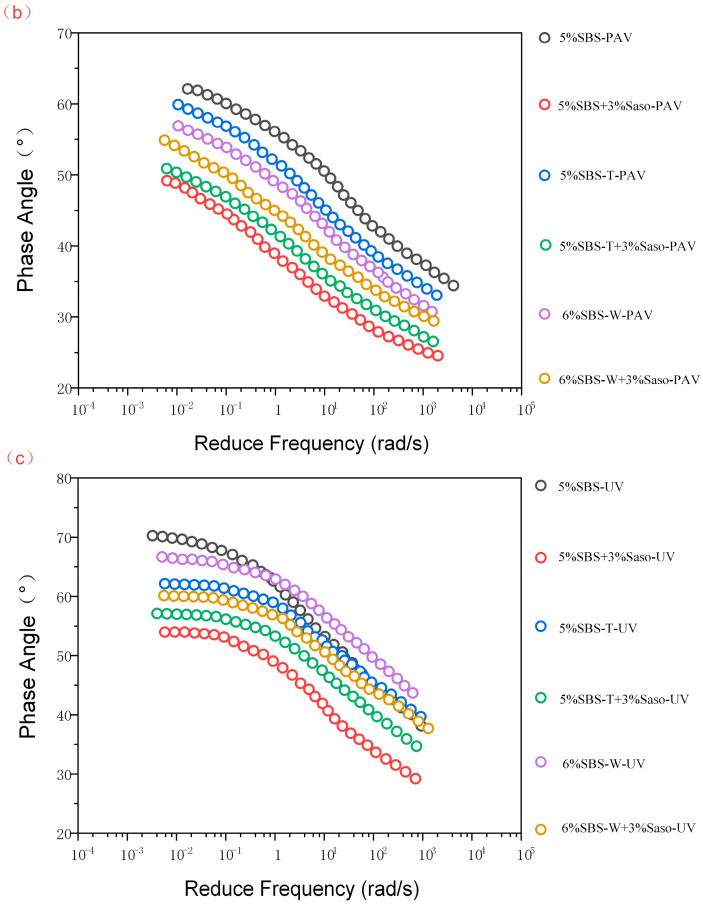
Phase angle main curve of modified asphalt: (**a**) Original asphalt, (**b**) PAV aging, and (**c**) UV aging.

The asphalt material’s loss modulus to storage modulus ratio is represented by the phase angle. It stands for the proportion of the asphalt’s elastic to viscous reaction. The more viscous the component of the modified asphalt, the higher the degree of the phase angle is. Rutting is a common result of simple deformation at high temperatures. The modified asphalt exhibited a more elastic reaction as the phase angle decreased. The actual project exhibited a higher deformation and rutting resistance. The following conclusions can be drawn from Figure 8:

(1) The phase angles of the six modified asphalt cements under four conditions tended to decrease with an increase in frequency, which was determined by the rheological properties of the asphalt materials. With the increase in frequency, the load time became shorter, and the asphalt deformation was mainly elastic deformation, which was shown in the data as a gradually decreasing phase angle [32].
(2) When the frequency was low, the phase angle of SBS-WMA was the largest. The phase angle value of the traditional SBS-modified asphalt was significantly larger than that of the quick-melting SBS-modified asphalt, indicating that the quick-melting SBS-modified asphalt could better withstand the fatigue damage caused by pressure aging and ultraviolet aging. With a gradual increase in frequency, the phase angle value of SBS-WMA dropped sharply and dropped to the lowest. It had a better deformation resistance and fatigue resistance under these two aging conditions.
(3) The main curves of the phase angles of the six as-is modified asphalt cements all had a plateau or peak. This phenomenon may have been caused by the physical crosslinking of polymer modifiers or the differences in the interactions (such as dispersion, expansion, and compatibility) between the polymer and asphalt molecules. The SBS modifier expanded in the base bitumen and absorbed the light component in the base bitumen, which caused the value of the phase angle to change.
(4) The asphalt binder changed by adding Sasobit proved much lower phase angle values and more viscous components than the asphalt binder without Sasobit. This behavior may suggest that Sasobit can greatly increase the deformation resistance of asphalt.

## 4. Conclusions

This study looked at the performance of warm-mixed fast-melting SBS-modified asphalt in order to increase the durability of asphalt pavement, while also taking into account the actual requirements of asphalt pavement material composition design. In addition, warm-mixed quick-melt SBS-modified asphalt’s modification mechanism, surface morphology, and thermal stability were examined using microscopic test methods (AFM and TG). The conclusions of this work can be summarized as follows:

(1) The “beehive” structure of the SBS-modified asphalt binder was finer and rougher, according to the AFM data. This was because the rapid-melting modifier helped to create a more solid network structure for the asphalt system while preventing the asphalt molecules from freely moving about and diffusing. It increased the modified asphalt’s resilience to fatigue.
(2) After the addition of the hot mix, the fast-melting SBS-modified asphalt still had a higher DMT modulus and adhesion value than the normal SBS-modified asphalt. This suggests that the quick-melting SBS modifier could enhance the stiffness and anti-deformation properties of the asphalt binder, resulting in improved anti-fatigue properties for the modified asphalt.
(3) The phase angle value of the fast-melting SBS-modified asphalt under the conditions of PAV aging and UV aging was significantly lower than that of the conventional SBS-modified asphalt, indicating that the fast-melting SBS-modified asphalt could better resist the fatigue damage caused by pressure aging and UV aging and had a better deformation resistance and fatigue resistance under these two aging conditions.
(4) The microscopic tests exhibited that the rapid-melting-type modifier encouraged the development of a more stable network structure in the asphalt system, increasing the modified asphalt’s resistance to fatigue. A high shear vibration was experienced by the methyl group in the SBS-T copolymer, and Sasobit could reduce the capacity of the polymer additives to absorb saturated hydrocarbons from the matrix asphalt. Fast-melting SBS-modified asphalt has a greater strength and crystallinity, making it more resistant to fatigue damage. A high thermal stability is also present in warm mix and fast-melting SBS-modified asphalt.

## Figures and Tables

**Table 1:** Technical information of base asphalt.

Parameters	Units	Values	Technical Requirement
Penetration test	0.1 mm	70	60–80
Softening Point test	°C	51.8	>46
Rotational viscosity (135 °C)	Pa·s	0.63	-
Ductility test	cm	>100	>100

**Table 2:** Technical information of modifiers.

Modifiers	Parameters	Units	Values
SBS	Oil content	%	0.70
	S/B ratio	-	30/70
	Total ash	%	0.20
	Tensile strength	MPa	18.0
	Volatility	%	1.00
	Elongation	%	700
Sasobit^®^	Viscosity at 135 °C	Pa·s	5.47 × 10^−3^
	Viscosity at 150 °C	Pa·s	3.26 × 10^−3^
	Flashing point	°C	290
	Melting point	°C	100
	Penetration at 25 °C	0.1 mm	1
	Penetration at 60 °C	0.1 mm	8

**Table 3:** Technical information of fast-melting modifiers.

Modifiers	Parameters	Units	Values
SBS-W	Appearance	Particle	-
	Individual weight	g	0.20
	Total ash	%	18.0
	Dry mix dispersibility	-	No particle residue
SBS-T	Appearance	Green particle	-
	Individual weight	g	0.25
	Total ash	%	0.42
	Dry mix dispersibility	-	No particle residue

**Table 4:** Roughness index of modified asphalt (nm).

Types of Modified Asphalt	Rms Roughness	Average Surface Roughness
5% SBS	3.68	1.53
5% SBS + 3% Saso	14.20	9.97
5% SBS-T	1.48	0.84
5% SBS-T + 3% Saso	9.08	6.74
6% SBS-W	5.56	3.28
6% SBS-W + 3% Saso	14.50	10.20

**Table 5:** Results of TG and DTG analysis of modified asphalt binders.

Asphalt Binders	IDT (°C)	T_max_ (°C)
5% SBS	392.19	471.53
5% SBS + 3% Saso	376.78	467.03
5% SBS-T	381.83	470.58
5% SBS-T + 3% Saso	378.44	471.44
6% SBS-W	379.44	472.42
6% SBS-W + 3% Saso	378.56	472.02

## Data Availability

Not applicable.
